# The Effect of Repetitive Transcranial Magnetic Stimulation on Motor Symptoms in Hereditary Spastic Paraplegia

**DOI:** 10.1155/2019/7638675

**Published:** 2019-05-12

**Authors:** J. Antczak, J. Pera, M. Dąbroś, W. Koźmiński, M. Czyżycki, K. Wężyk, M. Dwojak, M. Banach, A. Slowik

**Affiliations:** Department of Neurology, Jagiellonian University Medical College, Krakow, Poland

## Abstract

**Background:**

Hereditary spastic paraplegia (HSP) is a heterogeneous group of inherited disorders affecting predominantly the motor cortex and pyramidal tract, which results in slowly progressing gait disorders, as well as spasticity and weakness of lower extremities. Repetitive transcranial magnetic stimulation (rTMS) has been previously investigated as a therapeutic tool for similar motor deficits in a number of neurologic conditions. The aim of this randomized, controlled trial was to investigate the therapeutic potential of rTMS in various forms of HSP, including pure and complicated forms, as well as adrenomyeloneuropathy.

**Methods:**

We recruited 15 patients (five women and 10 men; mean age 43.7 ± 10.6 years) with the mentioned forms of HSP. The intervention included five sessions of bilateral 10 Hz rTMS over primary motor areas of the muscles of lower extremities and five sessions of similar sham stimulation.

**Results:**

One patient dropped out due to seizure, and 14 patients completed the study protocol. After real stimulation, the strength of the proximal and distal muscles of lower extremities increased, and the spasticity of the proximal muscles decreased. Change in spasticity was still present during follow-up assessment. No effect was observed regarding gait velocity. No changes were seen after sham stimulation. A post hoc analysis revealed an inverse relation between motor threshold and the change of the strength after active rTMS.

**Conclusions:**

rTMS may have potential in improving weakness and spasticity of lower extremities in HSP, especially of proximal muscles whose motor areas are located more superficially. This trial is registered with Clinicaltrials.gov NCT03627416.

## 1. Introduction

Hereditary spastic paraplegia (HSP) is a group of genetic disorders with slowly progressing degeneration of spinal motor pathways to lower extremities as a predominant pathologic feature. The key symptoms include impaired walking, muscle weakness, spasticity, hyperreflexia, and pyramidal signs as well as hypertonic bladder. Some genetic mutations associated with HSP predispose to additional neurologic deficits, such as cerebellar dysfunction, cognitive impairment, peripheral neuropathy, and extrapyramidal features, which determine the clinical differentiation between pure and complicated forms [1]. Due to a variety of genetic defects, pathophysiologic mechanisms in HSP may involve mitochondrial dysfunction, disturbances of lipid metabolism, axonal transport, myelination abnormalities, and a number of other mechanisms, which all result in the axonopathy of the corticospinal tract, which primarily affects the longest axons [2].

Adrenomyeloneuropathy (AMN) is another condition, which is phenotypically very similar to HSP and results from genetically caused impairment of peroxisomal *β*-oxidation of saturated straight-chain very long-chain fatty acids (VLCFA) [3]. AMN is commonly recognized as the form of adrenoleukodystrophy, a group of phenotypically different disorders caused by mutation in the ABCD1 gene. Parallelly, other researchers classify AMN as the x-linked, metabolic form of HSP [4, 5]. Similarly to HSP, biochemical impairment in AMN results in the axonal degeneration of the corticospinal tract with the longest axons being affected more severely and spastic paraparesis being the core symptom. In a subset of patients, peripheral neuropathy and cerebral demyelination may develop [6]. Currently, no disease-modifying therapy for any type of spastic paraparesis mentioned is available. Progressive weakness, spasticity, and walking impairment lead in many cases to wheelchair dependency and profoundly affect the quality of life [7].

Repetitive transcranial magnetic stimulation (rTMS) is a method of noninvasive modulation of brain activity, increasingly used in various psychiatric and neurologic conditions. Therapeutic benefits are related to induction of brain plasticity in disease-specific cortical areas, which is mediated by trains of magnetic pulses penetrating to brain tissue and depolarizing repetitively targeted neurons. According to previous data, magnetic pulses applied in high frequency (≥5 Hz) induce the long-term potentiation (LPT) with the increase of neural activity and excitability in the stimulated cortical area, whereas pulses in low frequency (≤1 Hz) suppress local neural activity by inducing the long-term depression [8]. A number of studies reported the beneficial effect of rTMS for weakness, spasticity, and gait impairment in conditions such as stroke, amyotrophic lateral sclerosis, multiple sclerosis, parkinsonism, and spinal cord injury [8–14]. Until now, this therapeutic option has not been investigated in any form of HSP. According to previous reports, pure and complicated forms, as well as AMN, are associated with decreased activity and reduced excitability of the motor cortex [15–18]. Therefore, we hypothesized that bilateral, high-frequency rTMS over primary motor areas for the muscles of lower extremities will improve the walking, muscle strength, and spasticity in patients with the mentioned types of HSP.

## 2. Methods

### 2.1. Participants

We recruited 15 patients (five women and 10 men; mean age 44.8 ± 10.1 years) with pure and complicated forms of HSP and with AMN. All of them suffered from chronic, slowly progressive spastic paraparesis with gait impairment being clinically manifested and according to patients' complaints significantly impairing daily life. Diagnosis was confirmed by genetic testing or by family history or was made by exclusion. Further inclusion criteria were the ability to walk 10 meters without or with crutches and age ≥ 18 years. Patients were excluded if they had one or more contraindications to rTMS listed in the safety guidelines issued by the International Federation of Clinical Neurophysiology (IFCN), i.e., magnetic material or electronic device in the body, history of epilepsy, and pregnancy [19]. Further exclusion criteria were cognitive impairment or psychiatric symptoms possibly interfering with the study procedure.

### 2.2. Study Design

We conducted a randomized, controlled trial in a crossover design. Investigations were made at the Department of Neurology, Jagiellonian University Medical College, Krakow, Poland. The study protocol was approved by the Ethics Committee of the Jagiellonian University (Permission No. 122.6120.119.2016). All participants gave their written informed consent. The study has been conducted in accordance with the Declaration of Helsinki.

### 2.3. Intervention

Before rTMS, every patient fulfilled a questionnaire for a TMS candidate based on the English original published by the IFCN [20]. Stimulation was done with the Magstim Rapid^2^ magnetic stimulator and with 110 mm double cone coil. 10 Hz rTMS was delivered over the bilateral primary motor area (PMA) of the muscles of lower extremities. The exact sites of stimulation and of estimating the motor threshold (MT) were the “hot spots” for the left and right abductor hallucis (AH). They were determined as the points on the scalp where the magnetic stimuli had produced the motor-evoked potentials (MEPs) of the highest amplitude. The recording electrodes were placed over the AH and over the proximal phalanx of the hallux. If no MEP could be recorded from AH, then one of the more proximal muscles of the lower extremity was chosen to determine the site of stimulation and to estimate MT. If no MEPs were evocable, rTMS was done with the coil placed over the vertex. Stimulation intensity for rTMS was set at 90% of the resting motor threshold (RMT) or, in case of prominent spasticity, when patient could hardly maintain the full muscle relaxation, 90% of the active motor threshold (AMT). MT was determined using the relative frequency method described in detail in the guidelines of the IFCN [21]. According to this method, MT is defined as the lowest intensity of magnetic field capable of evoking motor responses of certain amplitude after at least five out of ten stimuli. The amplitude of response required for estimation of RMT is ≥50 *μ*V, and the muscle should remain relaxed during examination. For AMT, the amplitude should be ≥200 *μ*V, and the muscle is slightly contracted, which in this study was achieved by asking the patient to perform a weak plantar flexion of the toes. In general, the rTMS protocol resembled the one applied by Kakuda et al. [22]. The real and sham stimulations contained five stimulating sessions, one a day, performed during consecutive working days. In every session, 1500 magnetic stimuli were delivered over PMA of the muscles of each lower extremity (3000 pulses per one session in total). The stimulation was made with 10 Hz frequency and in trains lasting 7.5 seconds (every train contained 75 stimuli, and there were 40 trains, per hemisphere, per session). Trains were separated with intervals lasting 56 seconds. During stimulation, participants were in a semirecumbent position and wore ear plugs, protecting them against the noise from the coil. The whole procedure was the same for the sham stimulation except that the coil was held perpendicularly to the scalp by rotating it posteriorly, by 90 degrees. This maneuver reduces the magnetic field by 67–73% making it devoid of any biological effects [23] but does not significantly change the clicking noise and sensations, which are similar to those of the active stimulation. In general, the sham procedure followed many previous studies with rTMS, including those with stimulation of motor areas of lower extremities [11, 22, 24]. Every included participant underwent real and sham stimulations in random order. The randomization list contained blocks of random size of two or four. Information about assignment of every patient was kept in sealed envelopes. Eight patients received the active treatment first. rTMS took place in the afternoon, usually between 2 p.m. and 4 p.m. The arms were separated in every patient by an interval lasting between one and three months. During participation in the study, patients continued their usual physiotherapy and medication for spasticity in an unchanged way. Most patients who took baclofen did it thrice a day with the second dose being taken about 1 p.m., i.e., before the rTMS session.

### 2.4. Assessment of Cortical Excitability and of Conduction in Central Motor Pathways

Before rTMS, the measurements of MEP amplitude, of the central motor conduction time (CMCT), and of the cortical silent period (CSP) were done. In accordance with the IFCN guidelines [21], MEPs were recorded five to six times during slight contraction of the target muscle (usually AH) after stimuli of 140-170% of RMT/AMT intensity. For the measurement, the MEP of the highest amplitude was chosen. The MEP amplitude was expressed in millivolts and as the percentage of the peripheral response, i.e., of the compound muscle action potential (CMAP) from the tibial nerve. CMCT was calculated using the previously published formula [21], utilizing the minimal F-wave latency. CMAP and F-wave were recorded according to the standard method used in neurography [25]. CSP was measured from five responses obtained during maximal voluntary muscle contraction after magnetic stimuli of the intensity of 140-170% of MT value, and the measurement followed the guidelines of IFCN [21]. MEP was considered abnormally low if its amplitude was less than 15% of CMAP [21]. The normal values of CMCT were adopted from other recommendations of IFCN [26].

### 2.5. Outcome Measurement

The primary endpoint was the change in the 10-meter walk test (10MWT) after rTMS and at follow-up. 10MWT measures the time a patient needs to walk 10 meters on a flat floor. The usual aids for walking should be used [27]. The alternative measure of gait performance used as the secondary endpoint was the timed up and go test (TUG). TUG is a complex measure of mobility. The subject starts with standing up from the chair of 45 cm height, walks three meters, turns around, walks back to the chair, and sits down. The time needed to perform is assessed [28]. Other secondary endpoints included the changes in the strength and spasticity of lower extremities after rTMS. The strength was measured with a microFET 2 hand-held dynamometer (Hoggan Scientific LLC, Salt Lake, USA) and spasticity with the Modified Ashworth Scale (MAS) [29]. Both tests included the following movements bilaterally: hip flexion, knee extension, knee flexion, ankle extension (dorsiflexion), and ankle flexion (plantar flexion). For every movement's strength tested in a given joint, the dynamometer was placed at a point located few centimeters proximally to the next distal joint (e.g., for testing the strength of the hip flexion, it was the point a few centimeters above the knee, and for the ankle extension, the point a few centimeters proximally to the metatarsophalangeal joint). The subject was instructed to execute the movement with his maximal strength through three seconds. During this time, the examiner resisted the examined subject's force, keeping the dynamometer in a constant position. To test the force of knee extension, the patient was sitting upright and the dynamometer was placed at the leg (above the ankle) hanging down, i.e., the knee was flexed 90°. The force of knee flexion was tested with the subject lying prone with the knee initially flexed 30°. Other movements were tested in the supine position. For every movement, the strength assessment was repeated thrice, and the arithmetic mean was taken as the final result. In case two consecutive measurements differed more than 10 Newtons in a particular movement, a fourth attempt was made, and the measurement which had shown the greatest difference with the others was excluded from consideration. MAS assess the spasticity in terms of resistance to passive movement. It is a six-point scale where zero indicates no spasticity and five indicates rigidity during passive movement, which is compatible with severe spasticity. (In comparison to the version of MAS which uses a 1+ grade, in our study, this grade meant grade 2, and grades 2, 3, and 4 meant in our study 3, 4, and 5, respectively.) All measurements were performed before the first session of each treatment arm, then directly (on the same day) after the last session, and finally two weeks later as a follow-up. Participants and investigators performing the assessment but not the person performing rTMS were blinded to the treatment arm. Also, the person analysing the datasets was not blinded. Outcome assessment was done in the afternoon, usually between 2 and 4 p.m.

### 2.6. Statistical Analysis

The measurements of spasticity and strength were averaged for both extremities in each movement tested, and then, the scores for movements of the proximal segments of lower extremities, i.e., hip flexion, knee extension, and knee flexion, as well as of the distal segments, i.e., ankle flexion and extension, were summarized. The times of performing 10MWT and TUG, as well as the spasticity and the strength of proximal and distal segments measured before active rTMS, were compared with respective measurements done after active rTMS and during follow-up. For sham rTMS, the same comparisons were done. Owing to the small number of subjects and the presence of ordinal data, the nonparametric Wilcoxon signed rank test was used. The significance level was set to *p* < 0.05. Power analysis was conducted in G∗Power v.3.2 software [30], for large (dz = 0.8), medium (dz = 0.5), and small (dz = 0.2) effect sizes, assuming a sample size of 15 subjects. The power for the three effect sizes was 80%, 56%, and 18%, respectively. The rest of calculations was done with the Statistica data analysis software system, version 12.0 (StatSoft, 2008; Palo Alto, CA, USA). Considering our interest in all symptoms tested (gait performance, weakness, and spasticity), which might respond to rTMS differently, as well as our intention to avoid excessive type II errors, which may occur in such a limited number of subjects, we decided not to conduct a correction for multiple comparisons.

## 3. Results and Discussion

One patient (male, 29 years of age) dropped out due to seizure. The remaining 14 patients completed the study. The seizure occurred during the third session of real stimulation, after about 1000 stimuli over the right PMA were elicited. It began with a tonic flexion of the hip and knee on the left side lasting several seconds. Then, a loss of consciousness, generalized convulsions, and lateral tongue bite followed. The seizure resolved spontaneously after two to three minutes from its onset. There was a postictal phase with confusion and drowsiness, which lasted 15 to 20 minutes. The patient was excluded from further stimulation. An electroencephalography performed on the next day was normal. Since then, the patient came several times to ambulatory control, and no sequelae occurred. Of other reported side effects, one female participant (62 years of age) complained about sleeplessness after the first two sessions of active stimulation. Several other participants complained about mild headaches during the first or the first and the second sessions of active stimulation. In one participant with a complicated form of HSP and atrophy of muscles of lower extremities (male, 38 years of age), responses from AH were below 50 *μ*V despite maximal stimulation, so estimation of MT was done with recordings from medial head of the gastrocnemius muscle (MHG). In another participant (male, 36 years of age), no response from any muscle was obtained, and the intensity of therapeutic stimulation was set to 70% of the maximal stimulator output. This patient did not present muscular atrophy, and the lack of MEPs was most probably due to affection of the corticospinal tract. In yet another participant (male, 41 years of age), the interhemispheric difference in MT value was 14% of the maximal stimulator output, so we decided to decrease the rTMS intensity over the hemisphere with higher MT (right), equalizing it to the rTMS intensity over the contralateral hemisphere. In three patients (including the patient who dropped out), stimulation intensity was derived from AMT (see Table 1). A technical complication, which occurred in several subjects who were stimulated with intensity of 65% of the maximum stimulator output or above, was the need to prolong the intervals between the several last trains in the session due to coil overheating. This prolongation did not exceed two minutes. The demographic and clinical data of recruited patients are presented in Table 1.

### 3.1. Assessment of Cortical Excitability and of Conduction in Central Motor Pathways

All patients showed abnormalities. The most common finding was the reduction of MEP amplitude, followed by prolongation of CMCT, which was present in all patients with AMN and in the majority of the others. CSP showed relatively fewer abnormalities. Detailed data are presented in Table 1.

### 3.2. Changes in Walking Speed, Muscle Strength, and Spasticity

After real stimulation, the strength of the proximal and distal muscles of lower extremities increased, and the spasticity of the proximal muscles decreased. Changes in spasticity were still present during follow-up assessment. No effect of rTMS was seen in 10MWT and TUG. No changes were seen after sham stimulation. Respective data are presented in Table 2.

### 3.3. Relation of Therapeutic Effect to Motor Threshold

**Figure 1:**
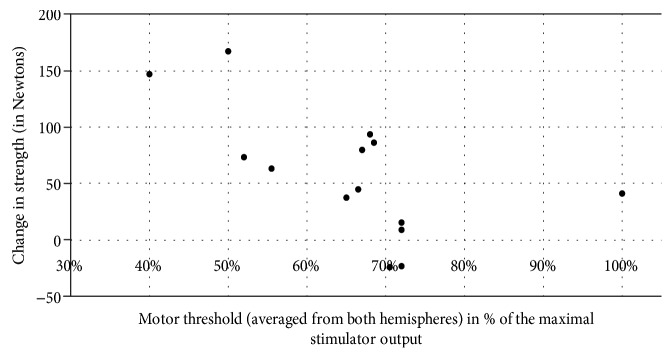
Relation of the motor threshold to the change in the strength of proximal muscles after real rTMS. *R* = −0.68, *p* = 0.008.

**Figure 2:**
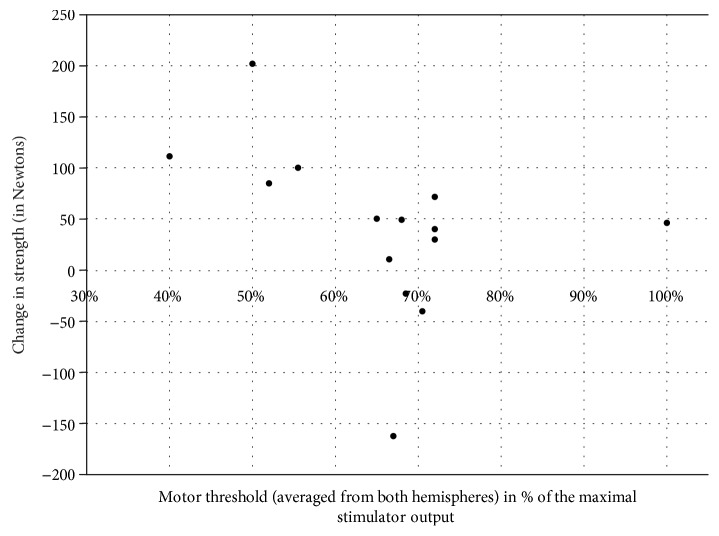
Relation of the motor threshold to the change in the strength of distal muscles after real rTMS. *R* = −0.57, *p* = 0.034.

Due to the big range of MT values in the studied group and resulting big range of rTMS intensities as well as previous findings, which linked changes in MT to HSP pathology [15], we carried out a post hoc analysis of the relation of MT to the therapeutic effect. The mean MT of 14 patients who completed the study was correlated with changes in strength and spasticity induced by active rTMS, which were significant, i.e., change in the strength of proximal and distal muscles after rTMS and change in spasticity of proximal muscles after rTMS and at follow-up. The MT value of the patient in whom no MEPs could be evoked was adjusted to 100% (of the maximal stimulator output). The change of strength in both, proximal and distal muscles, showed an inverse correlation with mean MT (*R* = −0.68, *p* = 0.008; *R* = −0.57, *p* = 0.034, respectively), whereas change in proximal spasticity showed no significant correlation (*R* = 0.38, *p* = 0.184 for change after rTMS; *R* = 0.03, *p* = 0.910 for change at follow-up). The relation between change in muscle strength and MT is presented in Figures 1 and 2.

### 3.4. Discussion

According to the authors' best knowledge, this is the first study which used rTMS for therapeutic purposes in HSP. The data indicate that rTMS may have potential regarding the strength and spasticity of lower extremities in this patient group. However, due to preliminary character, this study needs repetition on a bigger sample and with more sessions. Together with other studies using rTMS in acquired gait disturbances [9–11], as well as weakness and spasticity [8, 31], our work extends the evidence of the efficacy of noninvasive brain stimulation in therapy of these deficits. The key pathophysiological feature of the pure and complicated forms of HSP as well as of AMN is the retrograde axonal degeneration of the corticospinal tract [1, 32], which in the present study has been reflected by reduction of the MEP amplitude in almost all cases (in one case by a total absence of MEP). Therefore, observed changes in strength and spasticity may be associated with enhancement of excitability and metabolic activity in PMA and remaining corticospinal projections, which is a known effect of high-frequency rTMS [8] and which in our study may act similarly in all three types of paraparesis. At the cellular level, rTMS enhances BDNF-TrkB complex signaling and upregulates NMDA receptors, which induces synaptic plasticity, giving the possibility of remodeling of the neural circuits within central motor pathways [33]. Such remodeling results in changes of the resting-state functional connectivity as documented by functional magnetic resonance imaging on healthy subjects [34] and on patients with multiple sclerosis [35]. In both studies, rTMS over PMA, applied in excitatory protocols, decreased the connectivity between PMA and other brain areas, which was paralleled by the increase of MEP amplitude [34] and by reduction of spasticity in patients with multiple sclerosis [35]. These findings led us to suppose that a decrease of connectivity with other brain areas enhances the descending output of the motor cortex and improves the function of the pyramidal tract.

While the strength improved in both proximal and distal muscles, the effect on spasticity was seen only in the former ones. The lack of improvement in distal portions may be explained by the deeper localization of the respective primary motor areas, where the intensity of penetrating magnetic field is reduced. The increase of muscle strength and the reduction of spasticity were not paralleled by improvement in walking performance as measured by 10MWT and by TUG. The reason for this may lie in the complexity of gait disturbances in HSP, which not only results from weakness and spasticity but also involves other mechanisms such as disturbed sequence of the gait cycle due to abnormally high coactivation of antagonistic muscles [36] or abnormalities in spinocerebellar tracts [37]. In this light, it may be speculated that rTMS in combination with specific gait training could allow patients to implement the gains in strength and the decrease of spasticity to improve walking. Such a synergistic effect has been observed in patients after spinal cord injury who received rTMS and robot-assisted gait training [38]. In the present study, patients continued their usual physiotherapy, which probably did not influence the results. The second reason for lack of improvement in MWT and TUG may be the small sample size with resulting limited power of analysis. This is particularly true for MWT, where the *p* value was close to 0.05 when comparing measurements at the baseline and after rTMS.

Eight patients of our group received oral baclofen for spasticity. According to previous studies, this drug increases intracortical inhibition and reduces LTP-like plasticity in the human motor cortex [39, 40]. It is possible that pharmacotherapy interfered with the effect of rTMS and reduced observed improvement.

### 3.5. TMS Findings

Diagnostics with TMS showed abnormalities in all patients with the reduction of MEP amplitude being the most common finding. Similarly, the prolongation of CMCT was present in all forms of spastic paraparesis, including all patients with AMN. Of interest, one patient with HSP showed very pronounced prolongation of CMCT. This is in accordance with the study of Karle et al. [41] who documented such a prolongation in a small subset (6%) out of 128 patients with HSP and which may reflect myelination abnormalities associated with some of the genetic mutations leading to HSP. CSP showed similar values across diagnoses, which with few exceptions were normal and thus in line with the previous study done on patients with HSP caused by mutation of SPG42 [42]. The mean duration of CSP of our group was longer than values reported for the HSP linked to SPG4 mutation [15] but shorter than in patients with mutations in SPG4 and SPG7 genes as reported by another study [43]. The stronger magnetic stimuli used in our work in comparison to the former study and the measurement of CSP from the muscle of the upper extremity (first dorsal interosseous) in the latter may explain these discrepancies.

### 3.6. Relation of Therapeutic Effect to Motor Threshold

The lower MT correlated in our group with the bigger effect of real rTMS on muscle strength. Increased MT reflects among other variables the lower number and density of corticocortical connections and corticospinal axons [21]. According to this, we explain our finding by recognizing the low motor threshold as a marker of an early disease stage with still preserved plasticity and a high number of neural connections. Conversely, high motor threshold or complete lack of motor responses reflects severe loss of central motor neurons with only limited ability to respond to stimulation. Regarding the small number of subjects, this finding needs however repetition. Relation of motor threshold to the strength gain was in our study not paralleled by similar relation to the reduction of spasticity, but here, the limited number of degrees of freedom in the Ashworth scale may account for the lack of significant correlation.

### 3.7. Adverse Event

Even though stimulation was performed within the safety guidelines and even though our intervention was conducted similarly to previously published trials with rTMS over PMA of lower extremities [12, 14, 24, 44, 45], a seizure occurred. While conforming to the guidelines does not eliminate the risk of seizure completely, we suppose that the increased MT in HSP [15] and the resulting relatively strong therapeutic magnetic field might increase such risk. Further, we expect that estimating MT from the AH, as was performed in our study, might be another predisposing factor. Despite previous data showing that the AH has one of the lowest MT among the muscles of the lower extremity [26], we think that in some patients, MT for more proximal muscles, e.g., quadriceps or iliopsoas, may be lower than that for the AH due to a more superficial localization of the respective motor cortex. This notion is somewhat supported by the present results, which showed better reactivity to rTMS in proximal muscles. Therefore, determining the intensity of repetitive stimulation on the MT estimated in a single muscle might cause stimulation of certain cortical areas with greater intensity than desired. We suppose that estimation of MT from several muscles, including proximal and distal ones, may increase the safety of rTMS to lower extremities, especially in patients with HSP, who require a strong magnetic field for therapy. Another issue is the proximity of the motor cortices for the left and right legs. In case of a significant interhemispheric difference between MT values, the motor cortex contralateral to the stimulated one could theoretically receive a supramaximal stimulation. According to this, we consider that it may be beneficial for safety to adapt the intensity of therapeutic stimulation to the MT from the side where it is lower.

## 4. Limitations

The authors are aware of the preliminary character of the study and of limited power of the results. The evaluation of the datasets was done not blindly, which may be a potential source of bias. Another issue is the limited number of sessions, which might decrease the magnitude of the therapeutic effect and may be the reason why the improvement in strength was no more present in the follow-up. The cortical excitability has not been assessed after rTMS, which may limit the study value for optimization of rTMS protocols in future trials. Finally, the abundant pharmacotherapy with baclofen could considerably reduce observed effects.

## 5. Conclusions and Future Directions

Our study indicates that rTMS may have potential in improving the strength and spasticity of lower extremities in various forms of HSP. The proximal muscles of lower extremities may respond better to therapy due to the superficial location of respective cortices. The results warrant future studies, which should include therapy with more sessions and more subjects as well as monitoring of cortical excitability and neurophysiologic markers of spasticity. Further, the effect of rTMS should be investigated in conjunction with other therapies aiming at improvement of gait performance. Finally, as the studies on rehabilitation of gait with rTMS used different rTMS protocols, number of sessions, and coil types [9–11], the comparative studies are needed to optimize this kind of therapy. The seizure, which occurred, suggests rTMS over PMA of lower extremities may require specific precautions in patients with HSP.

## Figures and Tables

**Table 1:** Demographic and clinical data and TMS findings of recruited patients.

*N*	Gender	Age at incl.	Diagnosis	Dis. d.	Pharmacotherapy for spasticity	MT left	MT right	CMCT	MEP ampl.	CSP
1	M	38	AMN	13	Baclofen, tizanidine	74%	62%	22.4 ↑	0.8/10%	107
2	M	56	AMN	3		68%	66%	18.4 ↑	1.0/11%	280
3	M	41	AMN	14	Clonazepam, baclofen, and Lorenzo's oil	66%	78%	24.7 ↑	0.5/4%	122
4	F	42	Unspecified pure	8	Tizanidine	72%	69%	19.3 ↑	0.5/3%	119
5	M	38	Unspecified compl.	20		67% MHG	66% MHG	33 ↑	1.2/60%^∗^	129
6	M	29	HSP3A	26		57% AMT	53% AMT	14.7	1.4/10%	189
7	M	36	HSP7	8	Baclofen	No response	No response			
8	F	62	AMN gene carrier	6	Baclofen	72%	65%	18 ↑	2.4/13%	
9	M	54	Unspecified pure	17	Baclofen	57%	54%	16.8	0.6/7%	111
10	M	50	Unspecified pure	8		54%	50%	14.3	1.5/17%	229
11	M	53	Unspecified pure	8	Baclofen	50% AMT	50% AMT	12.8	0.4/7%	121
12	M	41	Unspecified pure	19		54% AMT	40% AMT	16.2	1.1/7%	132
13	F	46	AMN gene carrier	4		63%	67%	23.7 ↑	0.7/10%	93
14	F	48	AMN gene carrier	15	Baclofen	72%	72%	18.4 ↑	1.0/7%	119
15	F	22	Unspecified compl.	21		68%	76%	15	0.9/5%	109

F: female; M: male; age at incl.: age at inclusion; duration of sympt.: duration of symptoms (years); MT left: motor threshold of the left abductor hallucis (except subject 5) in % of the maximal stimulator output; MT right: motor threshold of the right abductor hallucis (except subject 5) in % of the maximal stimulator output; CMCT: central motor conduction time in milliseconds; MEP: motor-evoked potential (expressed in millivolts and as the percentage of the amplitude of respective peripheral response); CSP: cortical silent period (in milliseconds); AMN: adrenomyeloneuropathy; HSP: hereditary spastic paraplegia; MHG: medial head of gastrocnemius; compl.: complicated. ^∗^The unusually high amplitude of MEP in relation to peripheral response in this patient may be explained by atrophy of MHG, which decreased the peripheral response more profoundly than MEP, because MEP amplitude was probably a summation of potentials generated by MHG and adjacent muscles innervated by the peroneal nerve, which were located—due to atrophy—close to the recording electrode. Patient no. 6 dropped out. CSP was not done in patient number 8. As the published normative data for MT were done using circular coil [24], we did not assess MT regarding its normality.

**(a):** 

Real stimulation					
	Before rTMS	After rTMS	*p*	Follow-up	*p*
10MWT	21.90 ± 32.14	16.48 ± 16.37	0.074	16.05 ± 15.80	0.124
TUG	20.68 ± 25.98	15.96 ± 13.55	0.221	17.76 ± 19.31	0.158
Ashworth prox.	4.96 ± 3.29	3.29 ± 2.31	0.001	3.54 ± 2.81	0.018
Ashworth dist.	3.75 ± 2.07	3.64 ± 1.76	0.813	3.82 ± 1.92	0.612
Strength prox.	464.28 ± 190.74	522.15 ± 217.00	0.004	497.50 ± 196.37	0.198
Strength dist.	316.83 ± 174.41	357.78 ± 175.73	0.041	327.20 ± 159.25	0.510

**(b):** 

Sham stimulation					
	Before rTMS	After rTMS	*p*	Follow-up	*p*
10MWT	16.48 ± 14.49	18.18 ± 21.77	0.109	17.41 ± 19.83	0.300
TUG	17.83 ± 17.98	18.01 ± 20.39	0.433	18.60 ± 22.92	0.470
Ashworth prox.	3.68 ± 3.04	3.25 ± 2.52	0.508	3.14 ± 2.40	0.477
Ashworth dist.	3.39 ± 1.60	3.82 ± 2.03	0.236	3.36 ± 1.85	0.959
Strength prox.	467.02 ± 224.91	466.64 ± 187.19	0.975	452.70 ± 195.75	0.730
Strength dist.	272.39 ± 154.61	297.14 ± 154.41	0.158	294.84 ± 151.48	0.272

rTMS: repetitive transcranial magnetic stimulation; 10MWT: 10-meter walk test; TUG: timed up and go test; Ashworth prox.: spasticity score of proximal muscles; Ashworth dist.: spasticity score of distal muscles; strength prox.: strength of proximal muscles; strength dist.: strength of distal muscles (in Newtons).

## Data Availability

The scans of the sheets with noted results of TUG, 10MWT as well as the measurements of spasticity and strength used to support the findings of this study are available from the corresponding author upon request.
